# Spatial Genomics Identifies Heat Shock Proteins as Key Molecular Changes Associated to Adipose Periprostatic Space Invasion in Prostate Cancer

**DOI:** 10.3390/cancers17010002

**Published:** 2024-12-24

**Authors:** Olivier Cussenot, Lucie Poupel, Coralie Mousset, Julien Lavergne, Franck Bruyere, Alix Fontaine, Géraldine Cancel-Tassin, Gaelle Fromont-Hankard

**Affiliations:** 1 Department of Urology, Medical University of Vienna, 1090 Vienna, Austria; 2 CeRePP, 75020 Paris, France; 3 Nuffield Department of Surgical Sciences, University of Oxford, Oxford OX3 7DQ, UK; 4 Plateforme CHICS, Centre de Recherche des Cordeliers—UMRS 1138, 75006 Paris, France; 5 Inserm UMR 1069, 37032 Tours, France; 6 Department of Urology, Medical University of Tours, 37000 Tours, France; 7 Department of Pathology, Orleans University Hospital, 45000 Orléans, France

**Keywords:** prostate cancer, heat shock proteins, spatial transcriptomics, extra prostatic extension

## Abstract

Purpose: To identify molecular changes during PCa invasion of adipose space using Spatial Transcriptomic Profiling of PCa cells. Methods: This study was performed on paired intraprostatic and extraprostatic samples obtained from radical prostatectomy with pT3a pathological stages. Results: Differential gene expression revealed upregulation of heat shock protein genes: DNAJB1, HSPA8, HSP90AA1, HSPA1B, HSPA1A in PCa PanCK+ cells from the adipose periprostatic space. Extraprostatic extension was significantly associated with overexpression of genes involved in metastatic spread (EGR1, OR51E2, SPON2), of aggressiveness ERG negative signature of enhancers of androgen receptor (HOXB13, FOXA1), and of PSMA (FOLH1). They were associated with loss at 6q, 10q, 16q, and gain at 8q24 locus. Conclusions: PCa invasion of adipose EPE induces adaptative process related to heat shock proteins; PCa cells in EPE also present transcriptomics signatures for ERG independent aggressiveness, androgen receptor co-activation, and specific CNV changes.

## Simple Summary

Extraprostatic extension (EPE) of prostate cancer (PCa) cells in the adipose space (pT3a stage) is an unfavourable prognostic factor associated with recurrence and poor free metastatic disease-free survival outcomes of patients. However, molecular adaptation of PCa cells associated with EPE remains poorly understood. The aim of our research was to identify genomic determinants associated with EPE. We identified an overexpression of the heat shock protein genes: HSP90s, HSP70s, and HSP40s in PCa cells which infiltrate the adipose space. Moreover, we showed that EPE is also related to specific pathways associated with metastatic spread and enhancement of the androgen receptor pathway. These results provided valuable additional and new information to understand adaptative tumour changes during invasion of the adipose space This could be useful for the development of new markers or targeted therapies.

## 1. Introduction

Prostate cancer is one of the most diagnosed malignancies in men over 50 worldwide, with approximately 1.4 million new cases annually. It also accounts for nearly one-third of male cancer-related deaths in Europe [1]. An important difference in pathological staging lies between confined organ disease pT2 stage and pT3a stage, where the tumour extends beyond the gland margin (erroneously called “beyond the capsule”) [2]. McNeal et al. [3] showed that extraprostatic extension (EPE), usually called capsule penetration, correlated with the tumour volume, the location of positive surgical margins, and the presence of lymph node metastases or seminal vesicle (SV) invasion. Thus, stage pT3a is an unfavourable prognostic factor for cancer recurrence and poor metastasis-free survival [4]. In the pT3a stage, prostate cancer cells migrate into the pelvic adipose tissue surrounding the prostate. Adipose tissue is found in the vicinity of many invasive cancers. The adipose microenvironment can influence tumour behaviour via heterotypic tumour signalling processes through the release of free fatty acids, pro-inflammatory cytokines, or extracellular matrix proteins [5]. This underlines the concept that adipocytes participate in deleterious crosstalk with cancer cells to promote tumour remodelling, invasiveness, or drug resistance [6]. In prostate cancer, the molecular changes linked to the process of adaptation of the adipose environment are still unclear. It has been suggested that at the invasive front of the tumour, a lipolytic process occurs, leading to the transfer and accumulation of free fatty acids released into tumour cells [7]. These free fatty acids can increase oxidative stress and contribute to the activation of pathways involved in metastatic spread. Here, we analysed the molecular changes in tumour cells associated with infiltration of the periprostatic adipose space that may contribute to aggressiveness.

## 2. Materials and Methods

### 2.1. Patients and Tissues

Clinical cancer samples used for tissue microarray (TMA) construction were obtained from 27 PCa patients with extraprostatic extension (pT3) treated by radical prostatectomy at Tours University Hospital. To determine whether the expression of markers in cancer cells varies according to proximity of EPE, for each case, both the intraprostatic and the extraprostatic (cancer tissue in close contact with EPE) areas were sampled. Written informed consent was obtained from all patients following the requirements of the medical ethic committee of our institution.

### 2.2. TMA Construction

TMA was constructed using formalin-fixed paraffin-embedded tissue samples. For each patient, two areas of tumour tissue were selected, localised either within the prostate (intraprostatic area) or in extraprostatic area (PPAT). For each localization, three cores of 0.6 mm diameter were transferred from the selected areas to the recipient block, using a TMA workstation (Manual Tissue Arrayer MTA Booster, Alphelys, France).

### 2.3. GeoMx Digital Spatial Profiling

The GeoMx Digital Spatial Profiling (DSP) NGS technology (NanoString) that enables spatially targeted collection of oligonucleotide tags cleaved from specific validated target sequences was used to analyse the samples on the TMAs. Following the GeoMx DSP slide preparation manual (MAN-10150-02), TMAs were hybridised overnight at 37 °C with up to 18,000 barcoded photocleavable probes from the human whole transcriptome atlas. Morphological labelling using PanCytokeratin antibodies and SYTO13 guided the selection of a single region of interest (ROI) per spot. Oligonucleotides from the ROI were released via UV exposure, collected, and sequenced using Illumina Novaseq. Differential gene expression was analysed with GeoMx DSP software (https://nanostring.com/products/geomx-digital-spatial-profiler/geomx-dsp-overview/, accessed on 20 November 2024). The normalised data were used for downstream bioinformatics analyses.

### 2.4. Detection of Copy Number Variation (CNV) with Differential Gene Expression

Somatic copy number variations (CNVs) are strongly linked to the development and progression of numerous cancers. Pan-cancer studies established a close correlation between CNVs and differential gene expression at specific chromosomal loci (eQTLs) [8]. Using data from Kamoun et al., [9] which included both non-tumoural and tumoural samples analysed through CNV and transcriptomic arrays (Affymetrix HG U133 and Illumina Human CNV610 arrays, respectively), we selected a set of genes from the GeoMx^®^ gene list (https://nanostring.com/resources/geomx-human-whole-transcriptome-probe-list/, accessed on 20 November 2024) for their gene dosage imbalance (eQTL) linked to CNVs changes.

Our model predicted recurrent CNVs at specific loci (2q, 5q, 6q, 8p, 8q, 10q, 13q, 16q, 17p, 18q) with high accuracy (AUC > 0.84) based on eQTL data. Applying this model in this study, by comparing intraprostatic and extraprostatic tumours, allowed for identifying recurrent CNVs predominantly associated with EPE.

### 2.5. Statistical Analyses

Statistical analyses were performed using GeoMx DSP software and XLSTAT (XLSTAT statistical and data analysis solution. Lumivero, 2024. https://www.xlstat.com/fr, accessed on 20 November 2024). Comparisons between matched groups (intra- and extraprostatic zones from the same tumour) were performed using the matched Wilcoxon nonparametric test. Comparisons between independent groups were made using a non-parametric Mann–Whitney test (continuous data). A *p* < 0.05 was considered statistically significant for single tests. The Benjamani–Hochberg procedure was used to adjust the rate in differential expression analysis. Multiple comparisons were adjusted with Bonferroni correction. 

## 3. Results

**Figure 1:**
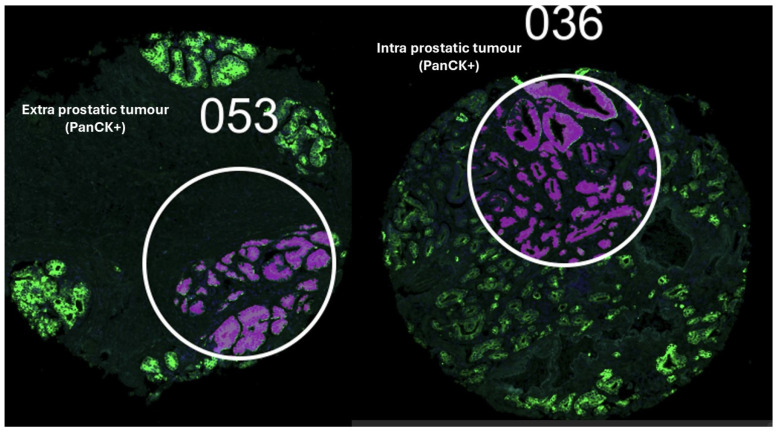
Extra and intraprostatic tumours labelled using PanCytokeratin antibodies and SYTO13 guided the selection of a single region of interest (ROI) per spot on the TMA.

From differential gene expression results, we focused on overexpressed genes in the extraprostatic groups. Using differential gene expression on PanCK+ cells enrichment analyses, we identified 11 genes that were the most dysregulated on the Volcano-plot. These genes belong to the heat shock protein (HSP) family (DNAJB1, HSPA8, HSP90AA1, HSPA1B, and HSPA1A) and are genes involved in metastatic spread (EGR1, OR51E2, SPON2), the prostate specific membrane antigen (PSMA) gene FOLH1, and two proto-oncogenes FOS and JUN (Figure 1 and Figure S1).

**Figure 2:**
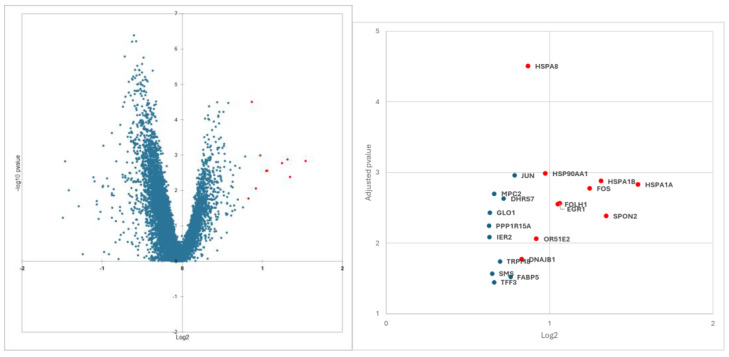
The 11 most overexpressed genes selected (red dot) on the volcano plot from differential gene expression analyses comparing extraprostatic and matched intraprostatic tumour tissues.

**Figure 3:**
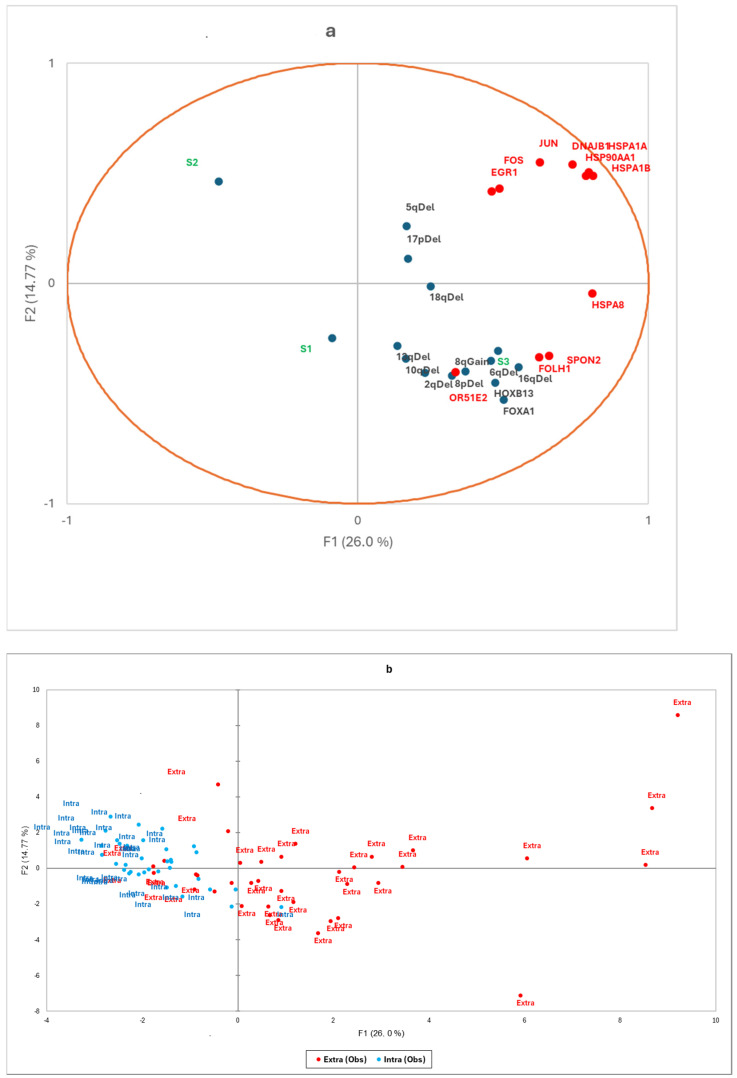
(**a**) Factorial analysis of mixed data and (**b**) factorial map of observations. The factorial analysis chart (**a**) illustrates correlations between the expression levels of the 11 candidate genes (DNAJB1, HSPA8, HSP90AA1, HSPA1B, HSPA1A, EGR1, OR51E2, SPON2, FOLH1, JUN, FOS) and transcriptomic signatures, including androgen receptor enhancers (HOXB13, FOXA1), ERG-dependent aggressiveness (S1, S2, S3), and eQTLs associated with copy number changes (2q, 5q, 6q, 8p, 10q, 13q, 16q, 17p, 18q deletions, and 8q Gain). The factorial map (**b**) shows the principal coordinates, highlighting extraprostatic tumours (in red) and intraprostatic tumours (in blue).

**Figure 4:**
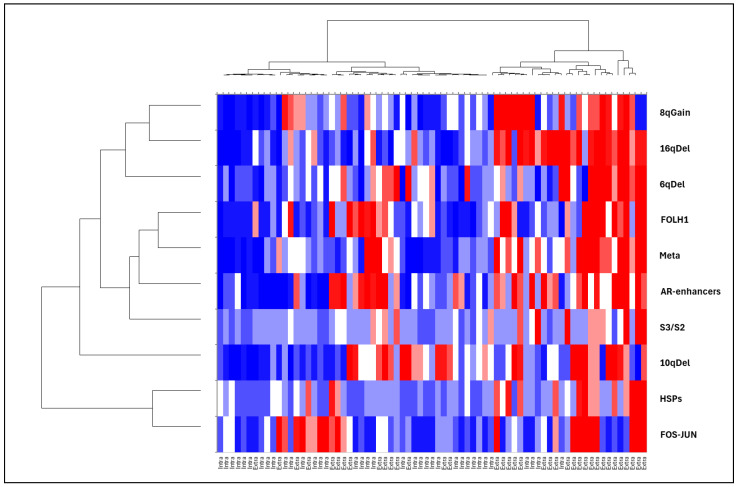
Heatmap displaying the mean expression of HSP genes, metastatic spread-related genes, PSMA (FOLH1), androgen receptor enhancer genes, and ERG-negative aggressiveness signatures (S3/S2). The heatmap also illustrates CNV changes at loci 16q, 6q, 10q, and 8q24, based on eQTL data. RNA expression differences were analysed using digital spatial genomics (PanCK+) to compare matched extraprostatic and intraprostatic tumour tissues.

Using the GeoMx^®^ gene set, we also analysed transcriptomic signatures known to be associated with PCa aggressiveness: expression of the androgen receptor (AR) and its enhancers (FOXA1, HOXB13), ERG-dependent aggressiveness signatures (classified as ERG-dependent aggressive (S1), ERG-independent aggressive (S3), and non-aggressive (S2) [9]), and eQTLs associated with recurrent copy number changes at loci 2q, 5q, 6q, 8p, 8q, 10q, 13q, 16q, 17p, and 18q (Supplementary Figure S2). Correlations with the 11 candidate genes identified in the Volcano plot are illustrated using a factorial analysis of mixed data (Figure 2) and a heatmap (Figure 3). Extraprostatic extension was significantly associated with the overexpression of androgen receptor enhancers (*p* < 0.001) and an increase in transcriptomic predictions of recurrent CNV losses at 6q (*p* < 0.001),16q (*p* < 0.001), and 10q (*p* < 0.001), as well as gains at the 8q24 locus (*p* = 0.001) (Figure S2). Correlations between the identified candidate genes and molecular signatures were plotted (Figure 3 and Figure 4) according to the extraprostatic or intraprostatic sample status.

## 4. Discussion

Prostate cancers that infiltrate the periprostatic adipose tissue (pT3a stage) are associated with an unfavourable prognosis, characterised by a higher risk of cancer recurrence and reduced metastasis-free survival [4]. Using NanoString GeoMx^®^ digital spatial transcriptomic profiling, we identified three main functional groups of genes associated with EPE. The first group includes stress proteins, specifically heat shock proteins (HSPs). Among the HSP families, we observed the overexpression of certain subtypes: HSP90s (HSP90AA1), HSP70s (HSPA8, HSPA1B, HSPA1A), and HSP40s (DNAJB1), categorised based on molecular weight. HSPs are associated with tumour progression and adverse clinical outcome. They provide malignant cells with a selective advantage through multiple functions, such as bypassing the cellular senescence programme, interfering with tumour immunity, promoting angiogenesis, and supporting metastasis. In PCa, elevated levels of HSP70s were previously demonstrated to be associated with higher Gleason scores, castration-resistant prostate cancer (CRPC), and significantly elevated serum levels in patients with PCa compared to those without PCa [10].

HSP70 and HSP40 interact with each other and both act as chaperones, stabilizing the inactive conformation of the androgen receptor (AR) [11]. Inhibition of HSP70s was shown to suppress PCa growth and to enhance the efficacy of anti-androgen therapies in CRPC, suggesting that HSP70 may serve as a predictor of androgen-dependent PCa [11]. Additionally, HSP70s and HSP90s play crucial roles in promoting epithelial-to-mesenchymal transition, invasion, migration, and metastasis in various cancers, including colorectal cancer, glioblastoma, breast cancer, and PCa [11]. Teng et al. [12] reported that reduced expression of HSP70s and HSP90s resulted in a loss of invasive potential in PCa. Given these insights, different strategies to inhibit HSPs are being explored in PCa drug development. For instance, monoclonal antibodies and aptamers have been proposed to target HSP70 expression, and synthetic inhibitors of HSP90 are under investigation [12].

The genes SPON2, EGR1, and OR51E1 were shown to be involved in pathways associated with metastatic progression [13,14,15]. SPON2 upregulation, which is linked to its promoter hypomethylation, was associated with enhanced bone metastasis in prostate and lung cancers. Similarly, EGR1 regulates angiogenic and osteoclastogenic factors, contributing to metastatic spread [14]. OR51E1, an orphan receptor, was reported to reduce prostate growth in PCa but paradoxically promotes invasiveness and metastasis [16].

In advanced and metastatic PCa, upregulation of the proto-oncogenes c-Jun and c-Fos was associated with poor prognosis and increased recurrence risk [17]. Clinical studies showed that higher levels of active c-Jun correlated with tumour growth that was resistant to castration, highlighting its role in aggressive disease progression.

In 2018, thanks to a multiomics analysis by Kamoun A. et al. [9], we identified three distinct molecular transcriptomic signatures associated with prostate cancer aggressiveness: aggressive ERG-dependent (S1), aggressive ERG-independent (S3), and non-aggressive (S2). The aggressive S1 and S3 forms exhibited different patterns of copy number variations (CNVs). S3 is enriched with deletions in 2q, 5q, and 6q, while S1 is characterised by ERG fusion with TMPRSS2 (21q deletion) and by more frequent 10q deletions. S2, in contrast, is associated with a lower number of CNVs. The two (S1 and S3) divergent evolutionary trajectories were recently confirmed by Woodcock DJ et al. [18] and referred to as canonical (S1) and alternative types (S3). In this study, we found that tumours infiltrating the extraprostatic space, in contrast to intraprostatic tumours, closely resemble the aggressive S3 molecular signature, lack the S2 signature, and show decreased expression of genes located at the 6q12-22 locus, including MAP3K7. Loss of MAP3K7 was linked to early PSA recurrence in prostate cancer [19] and was commonly associated with the absence of ERG-TMPRSS2 fusion. Furthermore, MYC amplification was associated with a higher Gleason grade, poor prognosis, and represented the second most commonly amplified region in CRPC [20].

Moreover, extraprostatic invasion was associated with increased expression of the prostatic marker FOLH1 (PSMA) and of two AR enhancer genes: HOXB13 and FOXA1, but not with AR expression itself. Aberrant transcription in prostate cancer is not driven solely by AR activity but through the collaborative action of AR enhancers like those of HOXB13 and FOXA1 genes, which modify chromatin structure to facilitate AR and other factors in accessing cancer-specific binding sites. In non-transformed prostate cell line, FOXA1, when co-expressed with HOXB13, reprograms genome-wide AR occupancy to an aggressive prostate tumour model [21]. Additionally, HOXB13 interacts with the hormone-independent AR variant, AR-V7, which is essential for the transactivation of AR-V7 target genes [22].

PSMA binding is widely used for staging prostate cancer through PET scans, as reported by Fourquet A et al., [23], and in the castration-resistant, late stage of the disease to deliver radioisotopes, such as lutetium or actinium, for treating PCa metastases [24]. We observed an increase in PSMA gene expression associated with EPE. These results are in agreement with previous studies demonstrating that AR inhibitors increased PSMA protein expression in PCa cells with initially low PSMA levels. These results were explained by the co-occupation of HOXB13 and AR in PSMA enhancers, with knock-out models indicating that HOXB13 is a positive upstream regulator of PSMA in AR-positive and AR-negative PCa [25].

Obviously, one of the limitations of this study is that the number of included patients was relatively small (27); therefore, it will be necessary to assess the prognosis value of the identified candidate genes in a large-scale study. The relationship between the adaptation of biological pathways of prostate cancer cells to the adipose microenvironment during extraprostatic extension [26,27] and their capacity for distant dissemination and resistance to castration or taxane-based chemotherapies are also potential avenues for further studies.

## 5. Conclusions

In this study, we demonstrate that heat shock proteins (HSPs) and genes promoting metastatic spread are overexpressed by tumour cells during adipose EPE. These findings align with adaptive metabolic changes tied to the tumour microenvironment, such as oxidative stress, hypoxia, and lipid abundance [28,29]. Additionally, we identify that EPE at the pT3a stage is predominantly associated with ERG-negative aggressive pathways and is driven by androgen receptor enhancers FOXA1 and HOXB13. Our research therefore lays the foundations for future investigations into the invasion biology of extraprostatic PCa, particularly for the HSP family, which could be highlighted as potential biomarkers and targets for new drug and vaccine therapies.

## Data Availability

Data supporting the reported results can be requested from progene@cerepp.org.

## Supplementary Materials

The following supporting information can be downloaded at https://www.mdpi.com/article/10.3390/cancers17010002/s1: Table S1: Clinical and pathological data related to TMA specimens obtained from radical prostatectomy of patients with stage pT3a prostate cancer that were used for digital spatial genomics PanCK+ differential expression between matched extra-prostatic and intraprostatic tumour cells. Figure S1. ((a) Comparison of Mmean level of RNA expression of HSP genes (DNAJB1, HSPA8, HSP90AA1, HSPA1B, HSPA1A) in between intraprostatic (blue) compartment and in extraprostatic (red) compartments (b) Comparison of mean level of RNA expression of genes involved in mMetastastic genes spread (EGR1, OR51E2, SPON2) in between intraprostatic (blue) and in extraprostatic (red) compartments. Figure S2. (a–f): Signatures from NanoString GeoMx^®^ Digital Spatial tTranscriptomic genes set. Expression of set of genes to analyse enhancers of androgen receptor activity (HOXB13, FOXA1), Prostate specific antigen PSMA gene (FOLH1), Aggressiveness ERG dependant (S1, S2, S3), and eQTL of recurrent copy number changes (2q, 5q, 6q, 8p, 10q, 13q, 16q, 17p, 18q deletions and 8q Gain).
