# Supplementary material for: Spatial Genomics Identifies Heat Shock Proteins as Key Molecular Changes Associated to Adipose Periprostatic Space Invasion in Prostate Cancer
Source: Cancers (Basel). 2024 Dec 24;17(1):2. doi: 10.3390/cancers17010002 (PMC11718861; doi:10.3390/cancers17010002)

Supplement Material

Table S1: Clinical and pathological data related to TMA specimens obtained from radical prostatectomy of patients with stage pT3a prostate cancer that were used for digital spatial genomics PanCK+ differential expression between matched extra-prostatic and intraprostatic tumour cells.

PSA before radical prostatectomy.	ISUP (Gleason score)	Age at radical prostatectomy (years)
20.7	ISUP 3 (4 +3)	72
13	ISUP 3 (4 +3)	60
4.26	ISUP 3 (4 +3)	67
8	ISUP 4 (4+4)	74
24	ISUP 5 (5+4)	67
8,5	ISUP 3 (4 +3)	65
12.5	ISUP 3 (4 +3)	74
24	ISUP 5 (5+4)	73
52	ISUP 5 (4+5)	56
8.15	ISUP 3 (4 +3)	64
6.4	ISUP 4 (4+4)	66
10.1	ISUP 3 (4 +3)	65
9.98	ISUP 3 (4 +3)	67
12.4	ISUP 5 (4+5)	68
13	ISUP 3 (4 +3)	74
11	ISUP 3 (4 +3)	60
26	ISUP 3 (4 +3)	52
21	ISUP 3 (4 +3)	69
16.38	ISUP 3 (4 +3)	58
10	ISUP 5 (4+5)	54
17	ISUP 4 (4+4)	70
11.66	ISUP 3 (4 +3)	75
11.8	ISUP 3 (4 +3)	76
25	ISUP 3 (4+3)	66
29.5	ISUP 5 (5+4)	59
6.2	ISUP 3 (4 +3)	71
14.6	ISUP 4 (4+4)	56

Figure S1. (a) Comparison of mean level of RNA expression of HSP genes (DNAJB1, HSPA8, HSP90AA1, HSPA1B, HSPA1A) between intraprostatic (blue) and extraprostatic (red) compartments (b) Comparison of mean level of RNA expression of genes involved in metastatic spread (EGR1, OR51E2, SPON2) between intraprostatic (blue) and extraprostatic (red) compartments.

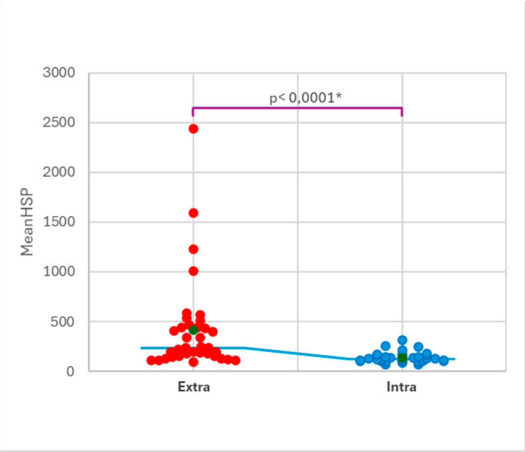

(a)

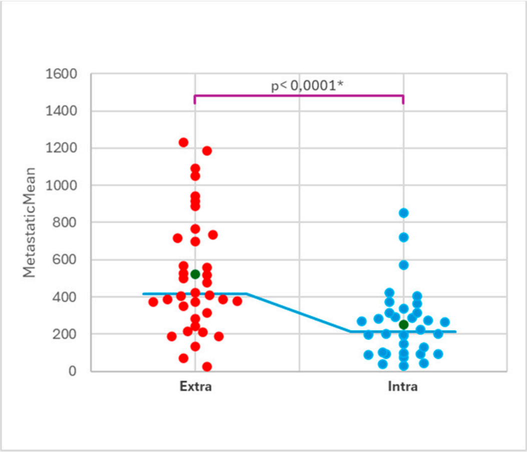

(b)

Figure S2 (a, b, c, d, e, f): Signatures from NanoString GeoMx® Digital Spatial transcriptomic genes set. Expression of set of genes to analyse enhancers of androgen receptor activity (HOXB13, FOXA1), Prostate specific antigen PSMA gene (FOLH1), Aggressiveness ERG dependant (S1, S2, S3), and eQTL of recurrent copy number changes (2q, 5q, 6q, 8p,10q,13q,16q,17p, 18q deletions and 8q Gain).

S2a: Comparison of mean RNA expression levelof AR enhancer genes (HOXB13, FOXA1) between intraprostatic (blue) and extraprostatic (red) compartments.

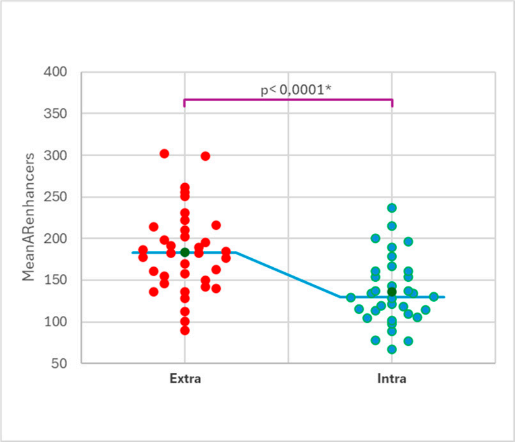

S2b: Comparison of mean RNA expression level of prostate-specific membrane antigen PSMA gene (FOLH1) between intraprostatic (blue) and extraprostatic (red) compartments.

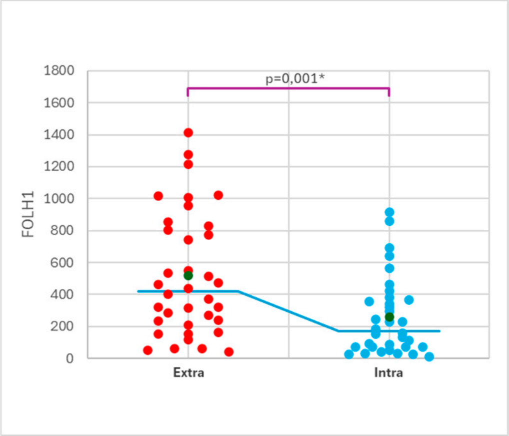

S2c: (i) Comparison of the ratio of (S3/S2 means) mean RNA expression level of the high aggressiveness ERG independent signature S3 (including expression of genesTFF3 ; RAMP1 ;SLC4A4 ;TPD52L1 ; EMC2 and HSD17B4 ; SLC1A1) and low aggressiveness signature S2 (: including expression of genes: SLC22A3 ; RRP1 ; RFPL2 ; PARM1 ; CPAMD8 ; PICK1 ; CATSPER2 ; FLNC ; SULT2B1 ; ZBTB20 and CSTF1) and (ii) comparison of ratio (S1/S2 means) of mean RNA expression of high aggressiveness ERG dependent signature S1 (including expression of genes: ERG ; TMEM45B ; TANC1 ; RGS17 ; LRRN1 ; SAMD1) and low aggressiveness signature S2, between intraprostatic (blue) and extraprostatic (red) compartments.

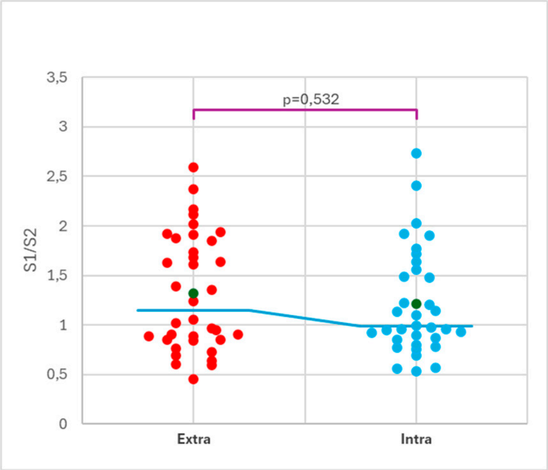

(i)

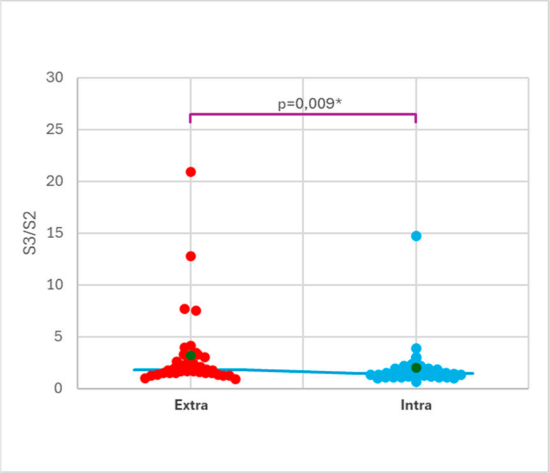

(ii)

S2d: Comparison of mean level of RNA expression of two proto-oncogens (Fos and Jun) between intraprostatic (blue) and extraprostatic (red) compartments.

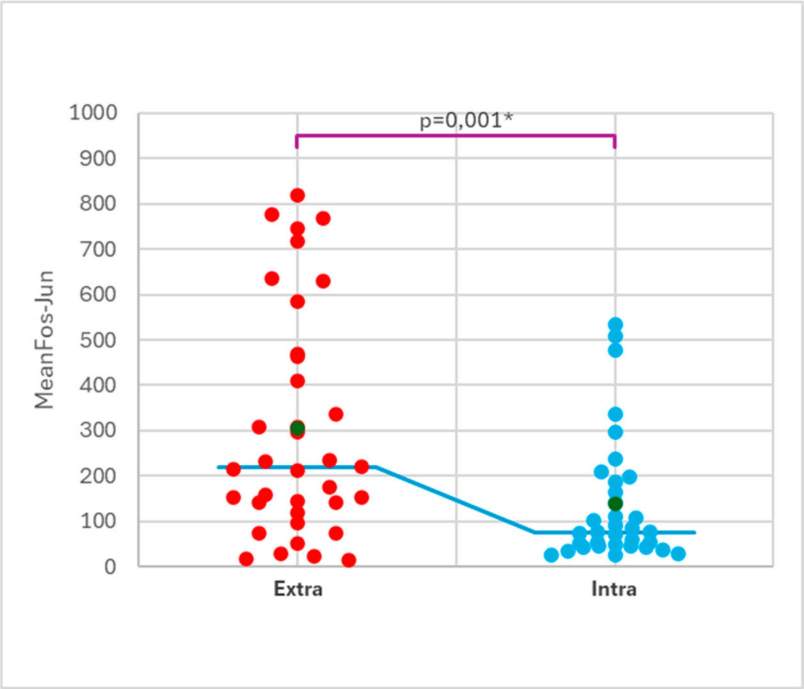

S2e: Comparison of 1/mean level (for deletion) of RNA expression of genes associated to CNV changes, between intraprostatic (blue) and extraprostatic (red) compartments.

-At 16q locus (genes: CDH3 ; IST1 ; MBTPS1 andBCAR1)

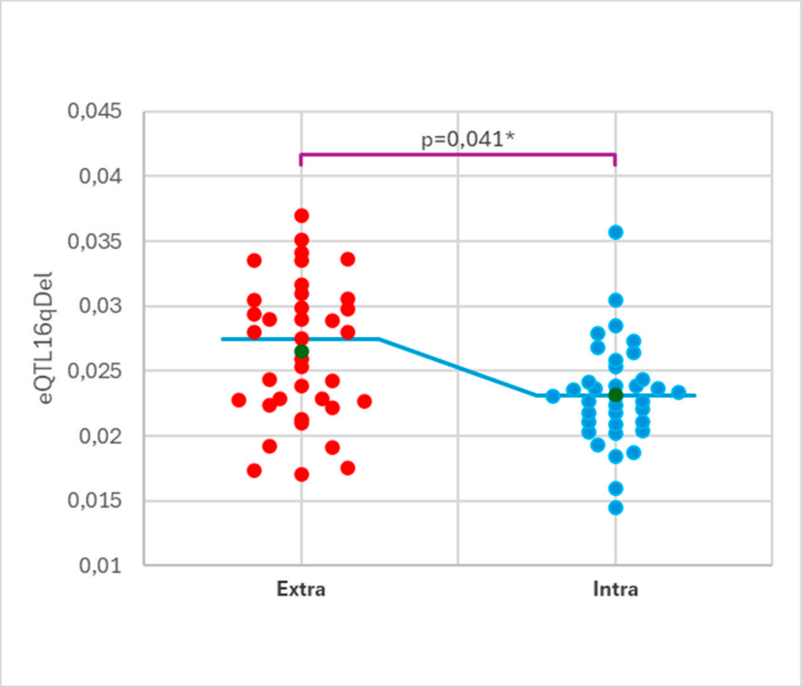

-At 8p locus (genes: XPO7 ; PPP3CC ; R3HCC1 ; TNFRSF10B ; PCM1 ; CHMP7 ; ELP3 ; LEPROTL1 ; EPHX2 and INTS10)

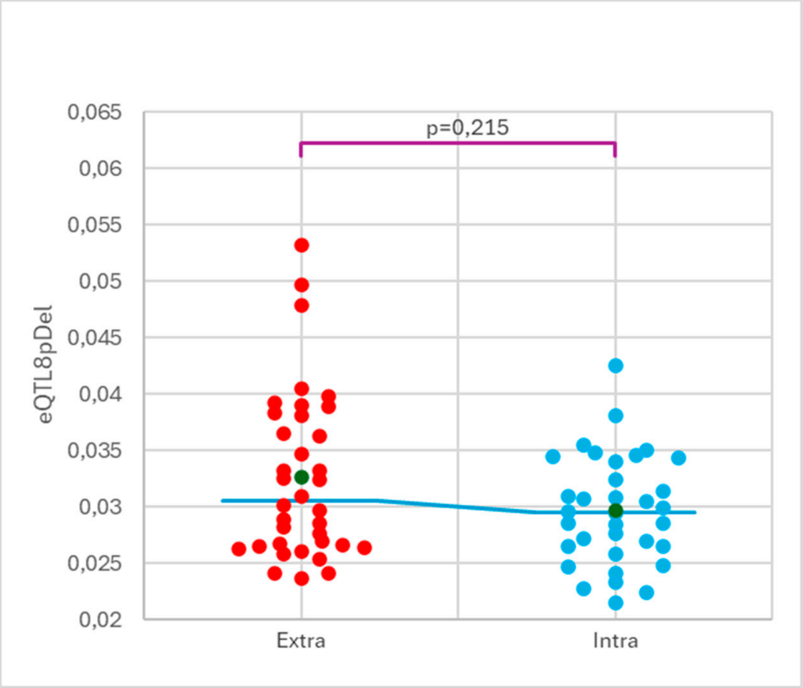

-At 10q locus (genes: ATAD1 ; TEP1 ; MYCBP2 ; PCGF5)

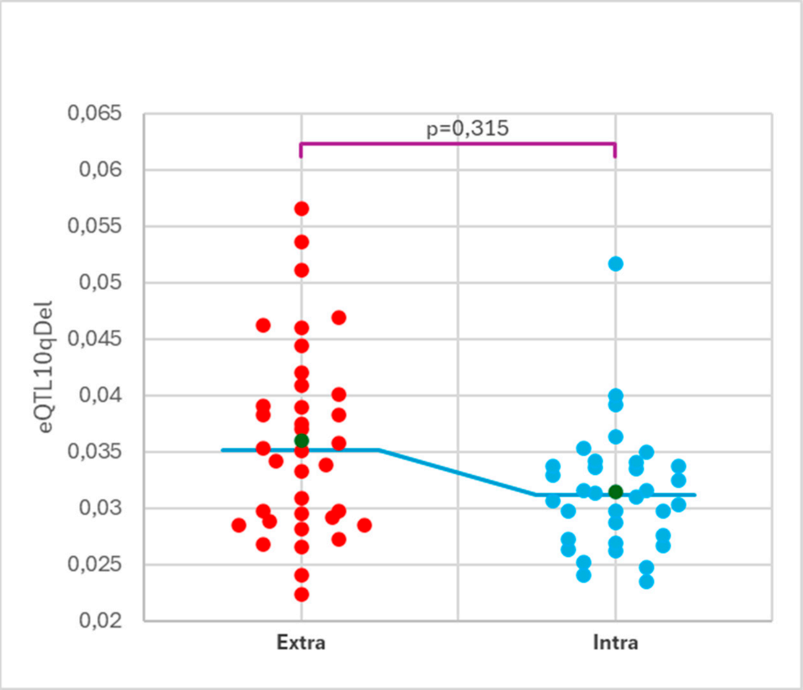

-At 13q locus (genes: FNDC3A ; MED4 ; LPAR6 ; RB1 ; ITM2B ; PHF11 andNAA16)

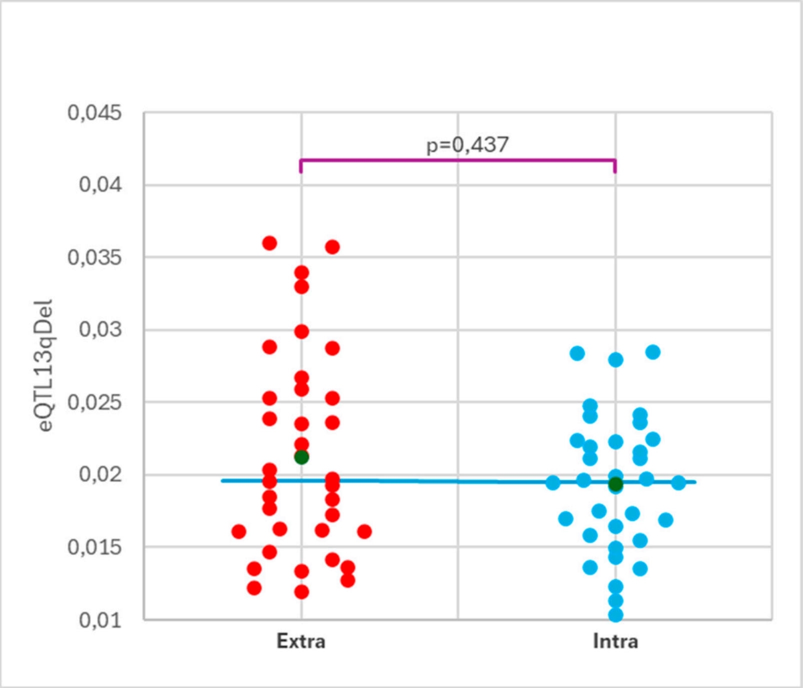

-At 17p locus (genes: ABR ; FXR2 ; ELAC2 and NCBP3)

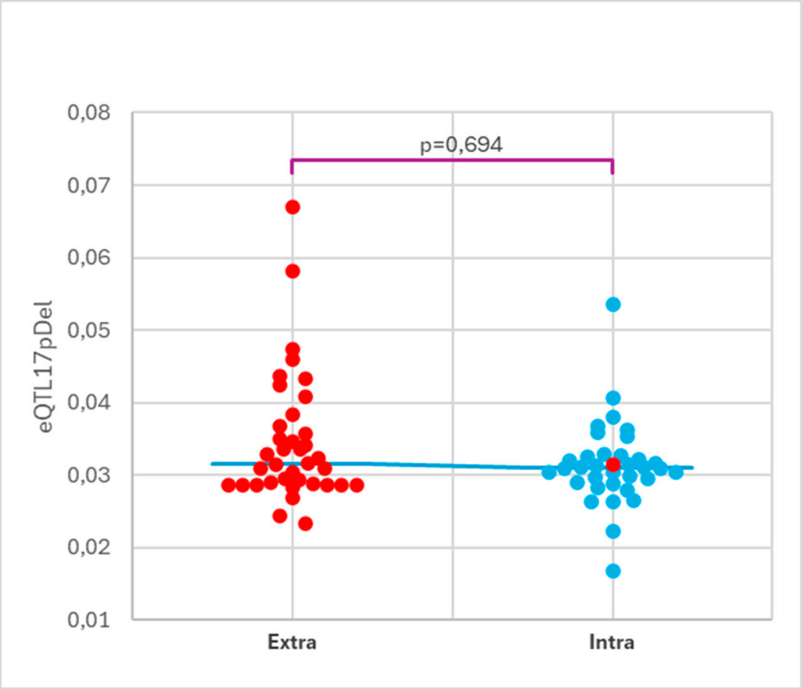

-At 6q locus (genes: MAP3K7 andRARS2)

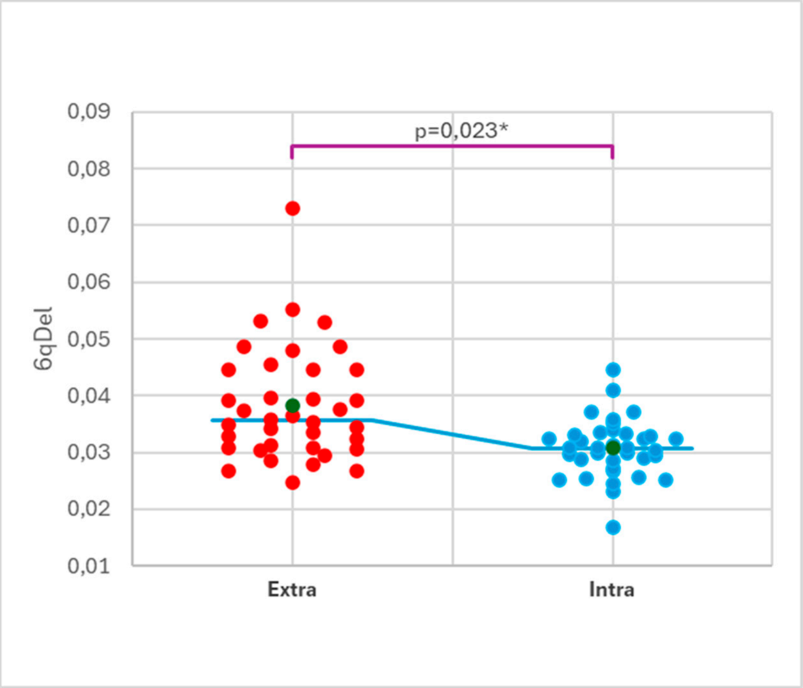

-At 5q locus (genes: CDK7 ; NSA2 andCDH1)

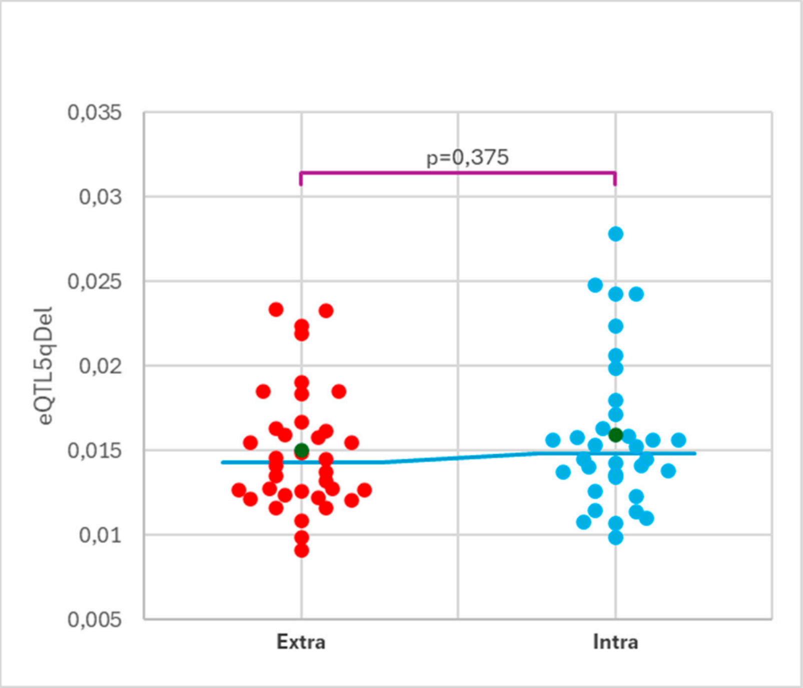

-At 2q locus (genes: SPOPL ; WDR33 andBIN1)

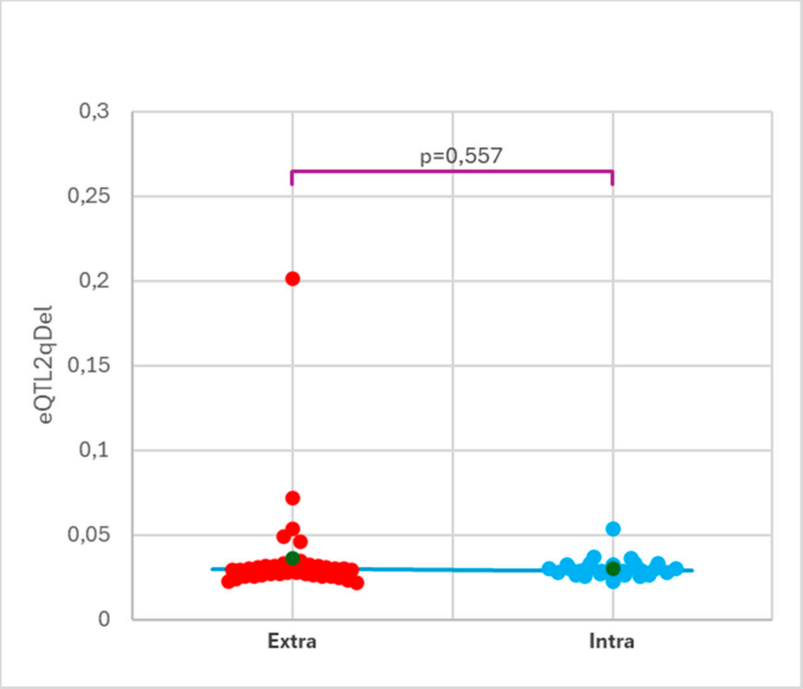

-At 18q locus (genes: ZCCHC2 and DYM)

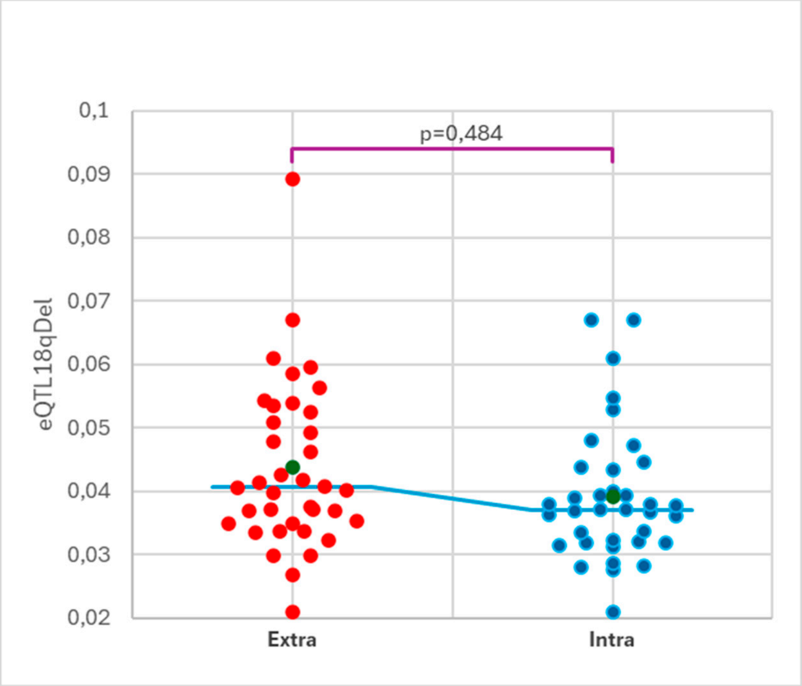

S2f: Comparison of mean level of RNA expression of genes associated to CNV changes between intraprostatic (blue) and extraprostatic (red) compartments at 8q locus (genes: TRIB1 ; MYC ;COPS5 ; WASHC5 ; DERL1 andYWHAZ).

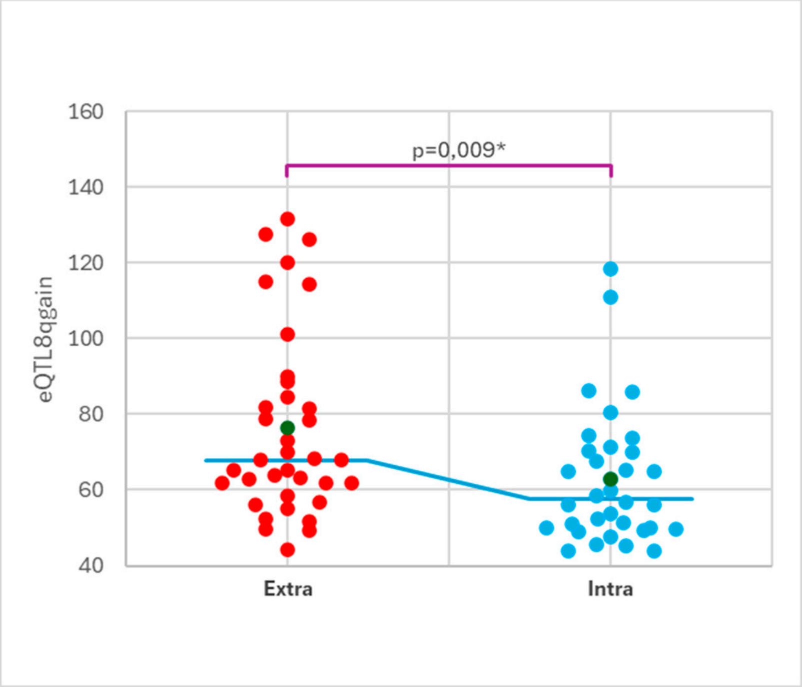

Supplement: Supplementary file 1 [file cancers-17-00002-s001.zip › cancers-3360155-supplementary.pdf]
